# Do point-of-care tests (POCTs) offer a new paradigm for the management of patients with influenza?

**DOI:** 10.2807/1560-7917.ES.2020.25.44.1900420

**Published:** 2020-11-05

**Authors:** Elizabeth M Dickson, Maria Zambon, Richard Pebody, Simon de Lusignan, Alex J Elliot, Joanna Ellis, Angie Lackenby, Gillian Smith, Jim McMenamin

**Affiliations:** 1 European Programme for Public Health Microbiology (EUPHEM), European Centre for Disease Prevention and Control (ECDC), Stockholm, Sweden; 2 Health Protection Scotland, Public Health Scotland, Glasgow, United Kingdom; 3 Reference Microbiology, National Infection Service, Public Health England, London, United Kingdom; 4 Centre for Infectious Disease Surveillance and Control, Public Health England, London, United Kingdom; 5 Nuffield Department of Primary Care, Oxford, United Kingdom; 6 Real-time Syndromic Surveillance Team, Field Service, National Infection Service, Public Health England, Birmingham, United Kingdom

**Keywords:** Influenza, point of care testing, opportunities, challenges, patient management

## Abstract

The introduction of point-of-care tests (POCTs) has presented new opportunities for the management of patients presenting to healthcare providers with acute respiratory symptoms. This Perspective article is based on the experiences of national infection teams/those managing acute respiratory infections across the United Kingdom in terms of the challenges and opportunities that this may present for public health. This Perspective article was conceived and written pre-coronavirus disease (COVID-19), however the principles we outline here for influenza can also be translated to COVID-19 and some key points are made throughout the article. The greatest challenge for intergrating POCTs into non-traditional environments is the capture of data and samples for surveillance purposes which provides information for public health action. However, POCTs together with measures outlined in this article, offer a new paradigm for the management and public health surveillance of patients with influenza.

## Background

Although point-of-care tests (POCTs) for influenza have been available for 20 years, the implementation of this technology in the United Kingdom (UK) has been slow due to problems with the sensitivity of the tests and how to integrate them into the care pathways. However, with the more recent expansion of the second generation nucleic acid amplification POC technologies, with improved sensitivity to the comparable ‘gold standard’ PCR laboratory tests, the implementation of these has become more acceptable in clinical settings.

While theoretically POCTs themselves could be performed at home, this application is not within the scope of this article. The definition of POCTs here is restricted to platforms with the potential to be used within 20 metres of patients and operated by a wide range of staff, including those without a laboratory background. The time to result may vary from 10 to 90 minutes [1]. This article will not cover the various tests that are available or diagnostic accuracies of these compared with the ‘gold standard’, as other published studies have covered this in depth [2,3].

From the literature, there has been successful use of the POCTs within hospital settings [4,5], paediatric emergency departments [6], community pharmacy settings [7] and outpatient departments [8]. This evidence and (more recent) experiences of others [9] suggest that there now may be an opportunity to change the way patients who present with acute respiratory symptoms are managed and to use POCTs as part of a healthcare pathway. This Perspective article aims to explore the opportunities and challenges of their introduction with a public health focus and provides an opinion on how they can be successfully and thoughtfully implemented into routine healthcare.

## Opportunities

Opportunity exists for the evaluation of the use of POCTs in primary care (Table). In the UK, there have already been moves towards the establishment of large primary care practices which could enable this targeted triaging of patients away from hospital. POCTs might influence the care pathways, providing reassurance that antibiotics are not needed and may create an opportunity for potential greater use of antiviral medication earlier in the course of the illness, thus maximising potential therapeutic effectiveness. POCTs may also enable the more timely use of antivirals in communal settings such as care homes, for example, where a rapid diagnosis of influenza can facilitate effective prescribing of antivirals based upon current National Institute for Health and Care Excellence (NICE) recommendations [10].

**Table:** Opportunities and challenges presented by influenza (and multiplex) POCTs for primary and secondary healthcare settings and public health

Settings	Opportunities	Challenges
**Primary and secondary healthcare**	• Enable targeted treatment in a timely manner reducing the risk of spread of influenza and other respiratory pathogens; • Early activation could provide data on vaccine effectiveness; • May assist with reduction of inappropriate antibiotic use in line with AMR strategy; • Allow greater segregation of acutely ill patients from those with chronic problems; • Reduce onward risk of transmission of influenza and other respiratory pathogens to others and reduced associated morbidity and mortality; • Allow release of single rooms through triaging patients in cohorts; • Potentially reduce length of hospital stay and nosocomial transmission of influenza and other respiratory pathogens; • Personalised medicine.	• Only large practices/practice federations likely to engage and need to be cost neutral/cost saving; • Integrating POCT process into clinical workflow (many of the POCT machines require 20 min operation time and GP consultation is 10 min); • Standardising protocols and harmonising technologies across authorities; • Higher demands on the services when patients present with viral illness to general practices or hospital front door; • Potentially increase transmission within community.
**Public health**	• Could increase testing of particular risk groups and generate better intelligence from surveillance; • Quicker time to result helping to inform action; • Improving data availability at community level.	• Capturing of data for surveillance from the laboratory systems; • Impact on surveillance data (proportion positives) and indicators (e.g. Goldstein for MEM) [20]; • Ensuring good quality surveillance data; • Reduced availability of samples for genetic and phenotypic testing in reference (public health) laboratories for analysis of strain distribution, vaccine effectiveness analysis.

AMR: antimicrobial resistance; GP: general practitioner; MEM: moving epidemic method; POCT: point-of-care test.

In the UK, the management of patients presenting with acute respiratory symptoms varies depending on the healthcare service they are presenting to, and which guideline(s) is/are being followed in each healthcare administrative region. However, the general principles are that (i) the patient will be triaged and, where appropriate, clinically assessed in community primary or secondary care, (ii) a presumptive diagnosis will be given (e.g. influenza-like illness) and if indicated, (iii) samples will be taken to be sent to the laboratory for confirmatory testing. The laboratory performs subtyping, sequencing and tests of antiviral susceptibility on all or subsets of samples and this information can subsequently be used by epidemiologists to follow disease trends, subtype and strain distribution and to provide estimates of vaccine effectiveness.

Dependant on clinical assessment, the patient will be sent home to recover or referred to hospital/admitted for further investigation and management with or without antibiotics/antivirals. The most obvious opportunity for the use of POCTs would be for more rapid triaging of patients at the hospital front door, putting them into appropriate care pathways thereby reducing the risk of onward transmission and consequent burden within hospitals and on the health service. Once at hospital, POCTs enable patient cohorting in bays of general wards or on designated influenza wards with reduced consequent risk of nosocomial transmission of influenza and improved patient flow [4]. On a larger scale, this could become the normal pathway associated with this group of patients in which POCTs may be cost saving by avoiding nosocomial hospital infections and ensuring appropriate targeted prescribing of antivirals/antibiotics. This latter may be of particular importance in an era of antimicrobial stewardship to minimise antimicrobial resistance. Post hoc analysis of a larger parent study has shown that reducing the turnaround time (TAT) of a test to less than 1.6 hours (such as those achievable by POCT), leads to a higher rate of early hospital discharge compared to longer TAT [5]. The authors surmise that this early discharge suggests that even a modestly more expensive diagnostic strategy is likely to be a cost saving compared to routine clinical care.

### Impact on antimicrobial treatment

The same post hoc analysis as mentioned above showed that the reduced TAT led to an earlier discontinuation of antibiotics compared with a longer TAT [5]. A large randomised controlled trial (RCT) in the UK was undertaken over two winter seasons in order to provide an insight into the clinical applicability of POC testing and the impact that it may have on a number of outcomes [6]. Although there was no reduction in the duration of antibiotics given overall, the patients in the POCT group received single doses or reduced courses of antibiotics compared with those in the control group, with a reduced length of stay, improved influenza detection and antiviral use. A more recent RCT from China measured the impact of POCTs for viral and atypical pathogens on intravenous antibiotic treatment duration in hospitalised adults with lower respiratory tract infection and saw a significant reduction in the duration of intravenous antibiotic treatment (p < 0.001) when POCTs were used [11].

While POCTs for influenza may increase antiviral treatment, their effect on morbidity and mortality are still being assessed [3].

### Personalised medicine

Recently, 46% of antibiotic prescriptions in England that could be mapped to a body system and/or clinical condition, were mapped to respiratory tract and ear, nose, throat infections [12]. Linked to this is a major seasonal variation in acute general practice consultations due to influenza and respiratory syncytial virus infections [13]. As mentioned earlier, some work has been done to assess the impact of POCTs on antibiotic use in secondary care [6,10]. However, to impact on antimicrobial prescribing, allow antibiotic sparing and contribute to reducing antimicrobial resistance, the appropriate and optimised use of antiviral agents needs to be evaluated [14,15]. An additional prospect of having antiviral resistance detection as part of POCTs would provide further rationale for appropriate clinical management of cases at the hospital front door (or earlier).

Vulnerable populations such as children and those aged 65 years or more are also worth considering when contemplating POCTs for personalised medicine. Children tend to have a higher viral load when infected with influenza which is advantageous for a POCT, however, those aged 65 years or more might have a reduced viral load and present late to care which may render the POCT falsely negative.

New potential third generation POCTs are in development which include host biomarkers as targets such as C-reactive protein as a non-specific marker for bacterial infection and the myxovirus-resistance A (MxA) protein, a derivative of interferon type I α/β which is indicative of the presence of a viral infection [16]. These tests offer another way for guiding treatment or stratifying management of presenting patients.

## Challenges

A number of assumptions are being made on the quality assurance and quality control of influenza POCTs and it may need to be clarified what their real-life sensitivity and specificity is. A study in a paediatric emergency department in Australia found that although a positive influenza POCT result led to a quicker diagnosis and reduced length of hospital stay, a negative POCT delayed diagnosis. The authors concluded that if influenza is still suspected, then further investigations should be performed to take account of the diagnostic uncertainty surrounding negative POCT results [17]. This can have important cost implications and delay the administration of antivirals, with negative therapeutic consequences.

### Capture of data and samples for surveillance

Integration of a new technology into clinical workflows is always challenging and may have unintended consequences. The principal challenge for integrating POCTs into non-traditional environments is the capture of data and samples for surveillance purposes to provide information for public health action. Current influenza surveillance relies upon the collection of data from multiple sources and the monitoring of individual surveillance components to provide a comprehensive record for analysis. The POCT results need to be captured by the Laboratory Information Management Systems (LIMS) to allow the inclusion of these data for surveillance.

Assuming high sensitivity and specificity (confirmed by local quality assurance of the testing systems used) there are residual important questions regarding data capture from POCTs. Do we get the timely results of positive tests and for the negative tests, do we get these results too (and thus may deduce the denominator and percentage positive)? Further, how do we deal with any step change in ascertainment bias from the widespread use of POCTs e.g. impact on the results of the laboratory positive or Goldstein/composite moving epidemic method [18,19] indicators? Finally, if POCTs only give an influenza A or an influenza B result, rather than H1N1, H3N2 subtype, etc. how do we get a representative picture of the circulating viral strains? This latter is particularly important to genetically characterise circulating influenza viruses and their relationship to the seasonal vaccine viruses, antiviral susceptibility and disease severity.

The experiences of a recent Scottish study undertaken during a season with increased pressure on hospital services from influenza A(H3N2) are illustrative of the practical problems for surveillance and short-term prediction based on the number of positive samples and the proportions of patients testing positive [20]. Our own observation is that the emergence of COVID-19 and the response to the global pandemic has led to an increased use of devices in non-standard environments such as schools. This means there will be more difficulties unless there is a concerted effort to assimilate the POCT results into routine reporting systems, requiring local system support.

## Discussion

In order to answer whether POCTs contribute to a new paradigm in the management of patients with acute respiratory symptoms, we first have to look at the opportunities and challenges that this technology presents. One of the main obstacles to evaluate the potential for influenza POCTs used outside the laboratory setting is the lack of published studies on the utilisation of the second generation nucleic acid amplification (PCR-based) technologies in clinical settings. The majority of POCT studies available to support this Perspective article were reporting on antigen tests which are known to have poorer sensitivity compared with the PCR-based tests.

Many aspects of influenza POCTs require to be addressed before implementation can be fully considered. Technological solutions, such as uploading data from POCT machines to cloud databases, or statistical techniques are available to overcome timely positive and negative tests as well as the ascertainment bias from widespread POCT use. The last one, obtaining information at influenza subtype level, may be addressed by a national policy to inform procurement. Consideration of the added benefit to surveillance of subtype data, as already outlined, can be justified in such a policy should the testing system be more expensive than that giving just influenza A or B result.

It has been shown that the potential benefit to patients and the healthcare systems that they present to may be considerable. The study by Youngs et al. suggests reduced number of hospital-acquired laboratory-confirmed influenza cases per day (0.66 cases vs 0.95 cases), a shorter median length of stay (5.5 days vs 7.5 days) and increased antiviral prescribing (80% vs 64.1%) [4]. In addition to this, the authors note that by cohorting the influenza-positive patients, trusts were able to collectively release 779 single rooms for use with other patients. The cost saving and opportunity created for alternative management of the freed resource may be substantial in each hospital particularly when scaled to a national basis. More studies are required on the cost-effectiveness of influenza POCTs in clinical settings in terms of clinical outcome and antibiotic use, as well as the more efficient use of isolation facilities resulting in reduced transmission and ultimately cost savings.

With influenza POCTs it is important to note that there is the further opportunity for taking the testing out of the laboratory and into non-traditional environments, e.g. care homes. There is already documented use of these tests in community pharmacies [7] which was shown to improve access to care as many patients visited outside clinic hours. This could potentially reduce the number of patients visiting out-of-hours, medical centres, emergency departments and hospital admission thereby reducing the number of exposure risks to other patients. On the back of this, there is an opportunity to investigate smart technologies that some devices coming on to the market now have, allowing results to be fed wirelessly into cloud-based systems for data capture. This may not yet be a feature of influenza POCT devices but is a likely direction of future development. With recent developments following the emergence of SARS-CoV-2, the COVID-19 National DiagnOstic Research and Evaluation Platform (CONDOR) is evaluating diagnostics in settings such as GP surgeries, care homes or hospitals, and accelerating how these technologies can be used in the real-world [21].

Given the experiences so far with COVID-19 and the overlap in symptoms with influenza, it is therefore vital that the distinction is made between these two serious infections and that rapid diagnosis is key. The ideal situation for the management of patients with acute respiratory symptoms would be that the POCT is performed early enough in the system to allow the patient to be triaged according to the test result and therefore minimising the subsequent exposure risks and potential for healthcare-associated infections. One of the key developments to come from the COVID-19 pandemic is that there is much greater interest in multiplex for several respiratory pathogens as was detailed by Brendish et al. prior to the pandemic [22]. POCT could therefore become much more informative for dealing with patients with severe respiratory symptoms. Indeed, Brendish et al. have since published on the use of POCT for the detection of COVID-19. Their evidence further supports the implementation of POCT into emergency departments and admission units prior to the next phase of the pandemic [23].

It is likely that POCTs could become part of a larger package for reducing influenza virus and SARS-CoV-2 infection challenges which would also include (i) hand washing policies, (ii) timely administration of antivirals and (iii) proper respiratory precautions when managing symptomatic patients. This should involve close scrutiny of health economic data on impact. The broader societal antimicrobial resistance agenda is also important to consider. The World Health Organization (WHO) global action plan on antimicrobial resistance includes the objectives to strengthen knowledge through surveillance and research, and optimise the use of antimicrobial agents [14]. These are key elements for the management of patients with influenza that we are proposing here.

The opportunities that are presented here with these new technologies are welcome. There will undoubtedly be many technology developments in the coming years to help meet the public health challenges and these need to be proactively adopted, with challenges worked through, to progress to improvement in healthcare delivery in different settings. The concurrent benefits of progress in digital technology and personalised methods should also be considered to bring in wider societal perspectives. It is our belief that POCTs taken together with the above measures offer a new paradigm for the management and public health surveillance of patients with influenza. In short, the potential of POCTs needs to be recognised and the existing ways of doing things need to be changed; all this will take time and careful handling.
